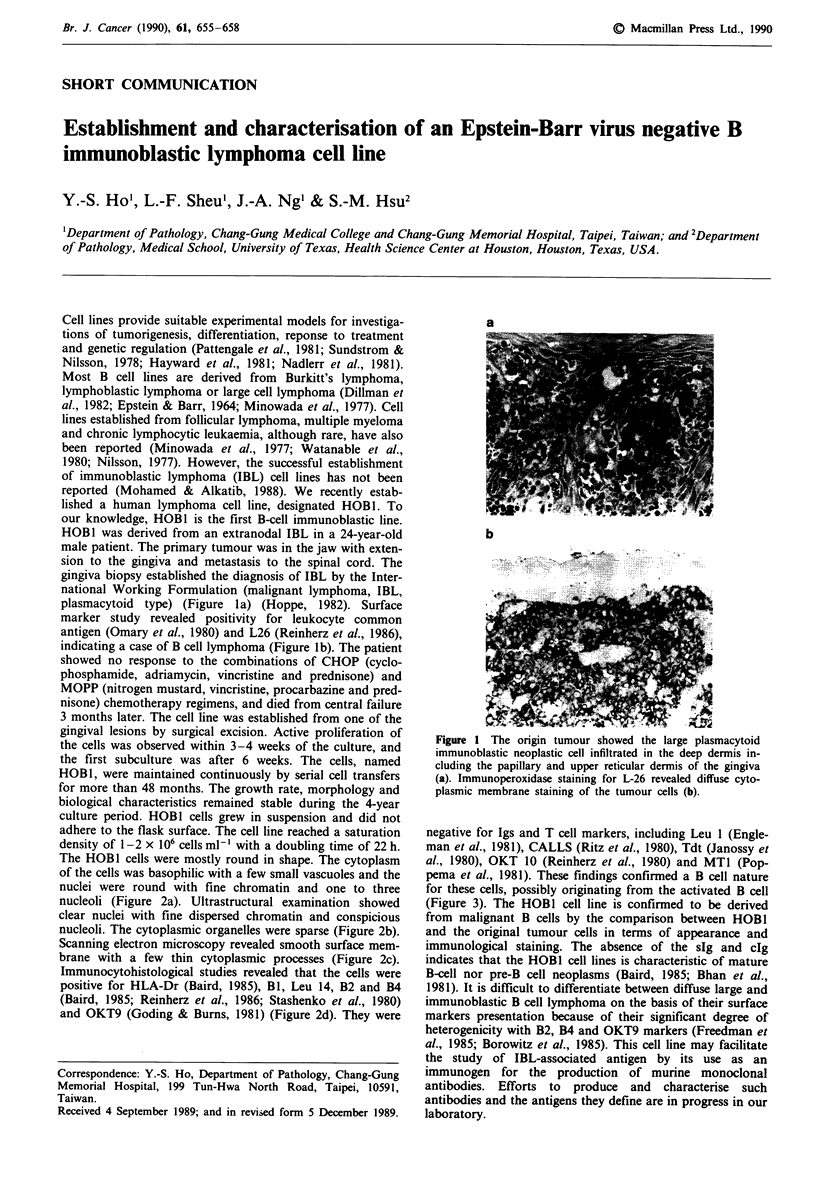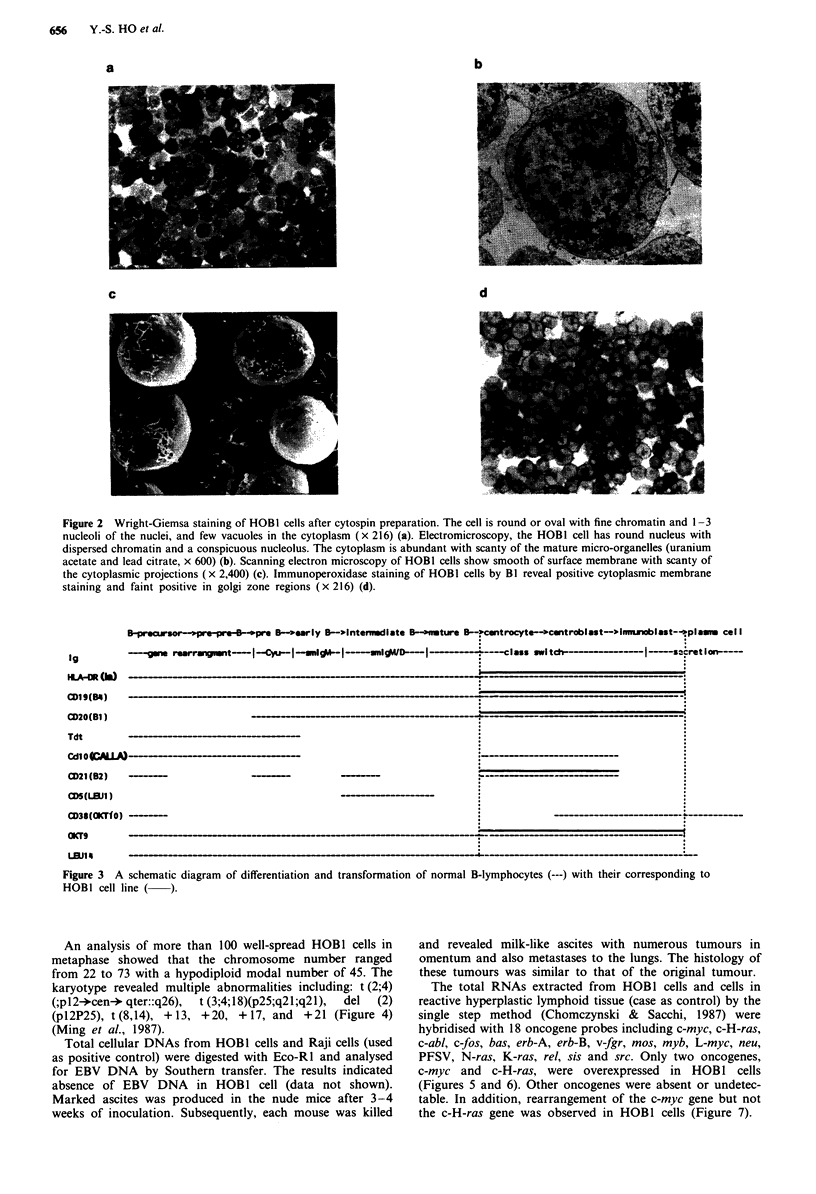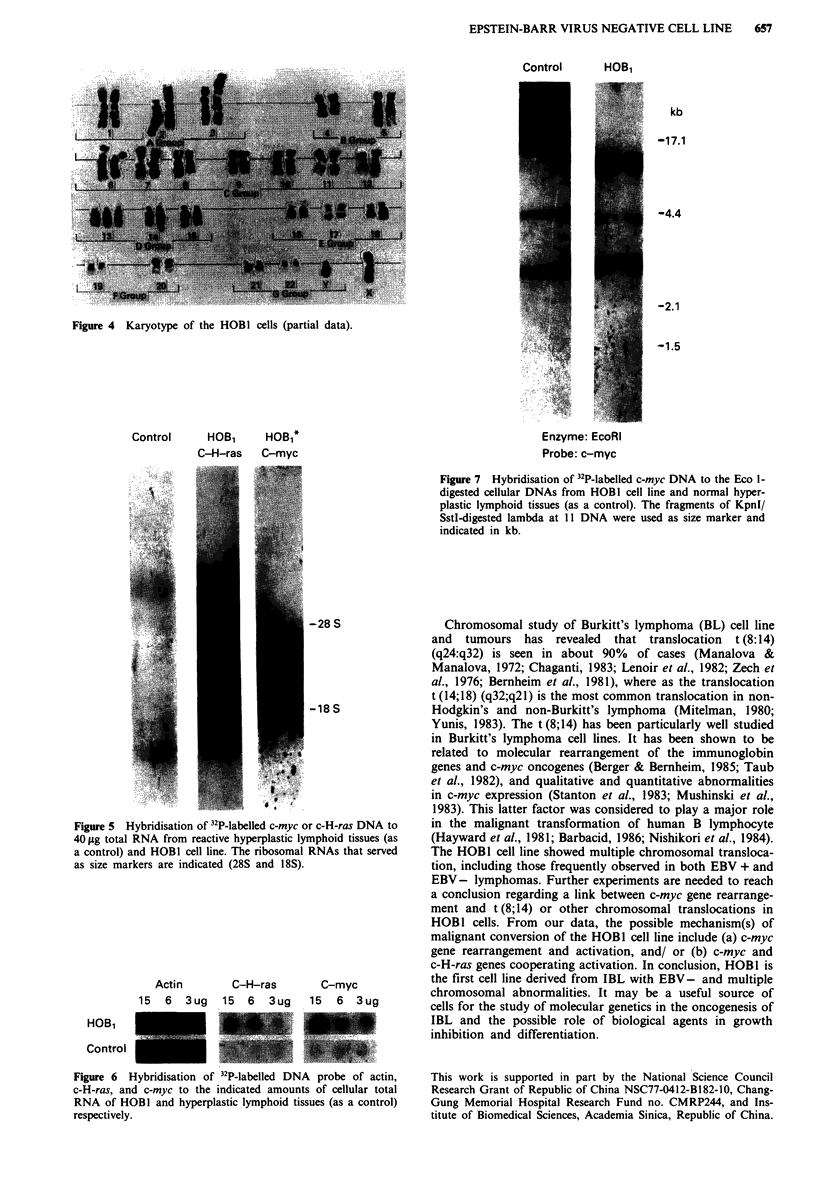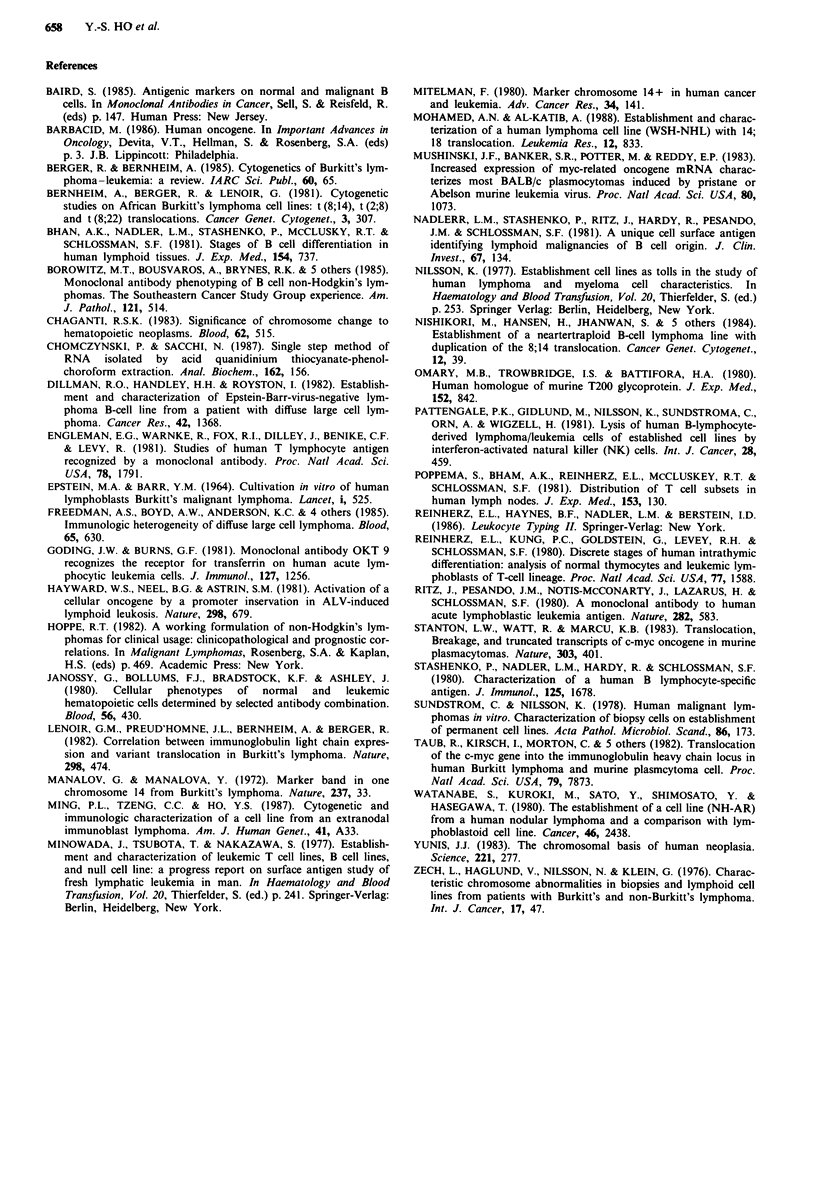# Establishment and characterisation of an Epstein-Barr virus negative B immunoblastic lymphoma cell line.

**DOI:** 10.1038/bjc.1990.148

**Published:** 1990-05

**Authors:** Y. S. Ho, L. F. Sheu, J. A. Ng, S. M. Hsu

**Affiliations:** Department of Pathology, Chang-Gung Medical College, Taipei, Taiwan.

## Abstract

**Images:**


Br. J. Cancer (1990), 61, 655-658 © Macmillan Press Ltd., 1990

SHORT COMMUNICATION

### Establishment and characterisation of an Epstein-Barr virus negative B immunoblastic lymphoma cell line

Y.-S. Ho[1], L.-F. Sheu[1], J.-A. Ng[1] & S.-M. Hsu[2]

[1]*Department of Pathology, Chang-Gung Medical College and Chang-Gung Memorial Hospital, Taipei, Taiwan; and* [2]*Department of Pathology, Medical School, University of Texas, Health Science Center at Houston, Houston, Texas, USA.*

Cell lines provide suitable experimental models for investigations of tumorigenesis, differentiation, reponse to treatment and genetic regulation (Pattengale *et al.*, 1981; Sundstrom & Nilsson, 1978; Hayward *et al.*, 1981; Nadlerr *et al.*, 1981). Most B cell lines are derived from Burkitt's lymphoma, lymphoblastic lymphoma or large cell lymphoma (Dillman *et al.*, 1982; Epstein & Barr, 1964; Minowada *et al.*, 1977). Cell lines established from follicular lymphoma, multiple myeloma and chronic lymphocytic leukaemia, although rare, have also been reported (Minowada *et al.*, 1977; Watanable *et al.*, 1980; Nilsson, 1977). However, the successful establishment of immunoblastic lymphoma (IBL) cell lines has not been reported (Mohamed & Alkatib, 1988). We recently established a human lymphoma cell line, designated HOB1. To our knowledge, HOB1 is the first B-cell immunoblastic line. HOB1 was derived from an extranodal IBL in a 24-year-old male patient. The primary tumour was in the jaw with extension to the gingiva and metastasis to the spinal cord. The gingiva biopsy established the diagnosis of IBL by the International Working Formulation (malignant lymphoma, IBL, plasmacytoid type) (Figure 1a) (Hoppe, 1982). Surface marker study revealed positivity for leukocyte common antigen (Omary *et al.*, 1980) and L26 (Reinherz *et al.*, 1986), indicating a case of B cell lymphoma (Figure 1b). The patient showed no response to the combinations of CHOP (cyclophosphamide, adriamycin, vincristine and prednisone) and MOPP (nitrogen mustard, vincristine, procarbazine and prednisone) chemotherapy regimens, and died from central failure 3 months later. The cell line was established from one of the gingival lesions by surgical excision. Active proliferation of the cells was observed within 3-4 weeks of the culture, and the first subculture was after 6 weeks. The cells, named HOB1, were maintained continuously by serial cell transfers for more than 48 months. The growth rate, morphology and biological characteristics remained stable during the 4-year culture period. HOB1 cells grew in suspension and did not adhere to the flask surface. The cell line reached a saturation density of $1-2 \times 10^6$ cells $ml^{-1}$ with a doubling time of 22 h. The HOB1 cells were mostly round in shape. The cytoplasm of the cells was basophilic with a few small vascuoles and the nuclei were round with fine chromatin and one to three nucleoli (Figure 2a). Ultrastructural examination showed clear nuclei with fine dispersed chromatin and conspicious nucleoli. The cytoplasmic organelles were sparse (Figure 2b). Scanning electron microscopy revealed smooth surface membrane with a few thin cytoplasmic processes (Figure 2c). Immunocytohistological studies revealed that the cells were positive for HLA-Dr (Baird, 1985), B1, Leu 14, B2 and B4 (Baird, 1985; Reinherz *et al.*, 1986; Stashenko *et al.*, 1980) and OKT9 (Goding & Burns, 1981) (Figure 2d). They were negative for Igs and T cell markers, including Leu 1 (Engleman *et al.*, 1981), CALLS (Ritz *et al.*, 1980), Tdt (Janossy *et al.*, 1980), OKT 10 (Reinherz *et al.*, 1980) and MT1 (Poppema *et al.*, 1981). These findings confirmed a B cell nature for these cells, possibly originating from the activated B cell (Figure 3). The HOB1 cell line is confirmed to be derived from malignant B cells by the comparison between HOB1 and the original tumour cells in terms of appearance and immunological staining. The absence of the sIg and cIg indicates that the HOB1 cell lines is characteristic of mature B-cell nor pre-B cell neoplasms (Baird, 1985; Bhan *et al.*, 1981). It is difficult to differentiate between diffuse large and immunoblastic B cell lymphoma on the basis of their surface markers presentation because of their significant degree of heterogenicity with B2, B4 and OKT9 markers (Freedman *et al.*, 1985; Borowitz *et al.*, 1985). This cell line may facilitate the study of IBL-associated antigen by its use as an immunogen for the production of murine monoclonal antibodies. Efforts to produce and characterise such antibodies and the antigens they define are in progress in our laboratory.

**Figure 1** The origin tumour showed the large plasmacytoid immunoblastic neoplastic cell infiltrated in the deep dermis including the papillary and upper reticular dermis of the gingiva (**a**). Immunoperoxidase staining for L-26 revealed diffuse cytoplasmic membrane staining of the tumour cells (**b**).

Correspondence: Y.-S. Ho, Department of Pathology, Chang-Gung Memorial Hospital, 199 Tun-Hwa North Road, Taipei, 10591, Taiwan.

Received 4 September 1989; and in revised form 5 December 1989.

**Figure 2** Wright-Giemsa staining of HOB1 cells after cytospin preparation. The cell is round or oval with fine chromatin and 1–3 nucleoli of the nuclei, and few vacuoles in the cytoplasm (× 216) (**a**). Electromicroscopy, the HOB1 cell has round nucleus with dispersed chromatin and a conspicuous nucleolus. The cytoplasm is abundant with scanty of the mature micro-organelles (uranium acetate and lead citrate, × 600) (**b**). Scanning electron microscopy of HOB1 cells show smooth of surface membrane with scanty of the cytoplasmic projections (× 2,400) (**c**). Immunoperoxidase staining of HOB1 cells by B1 reveal positive cytoplasmic membrane staining and faint positive in golgi zone regions (× 216) (**d**).

**Figure 3** A schematic diagram of differentiation and transformation of normal B-lymphocytes (---) with their corresponding to HOB1 cell line (——).

An analysis of more than 100 well-spread HOB1 cells in metaphase showed that the chromosome number ranged from 22 to 73 with a hypodiploid modal number of 45. The karyotype revealed multiple abnormalities including: t (2;4) (;p12→cen→ qter::q26), t (3;4;18)(p25;q21;q21), del (2) (p12P25), t (8,14), +13, +20, +17, and +21 (Figure 4) (Ming *et al.*, 1987).

Total cellular DNAs from HOB1 cells and Raji cells (used as positive control) were digested with Eco-R1 and analysed for EBV DNA by Southern transfer. The results indicated absence of EBV DNA in HOB1 cell (data not shown). Marked ascites was produced in the nude mice after 3–4 weeks of inoculation. Subsequently, each mouse was killed and revealed milk-like ascites with numerous tumours in omentum and also metastases to the lungs. The histology of these tumours was similar to that of the original tumour.

The total RNAs extracted from HOB1 cells and cells in reactive hyperplastic lymphoid tissue (case as control) by the single step method (Chomczynski & Sacchi, 1987) were hybridised with 18 oncogene probes including c-*myc*, c-H-*ras*, c-*abl*, c-*fos*, *bas*, *erb*-A, *erb*-B, v-*fgr*, *mos*, *myb*, L-*myc*, *neu*, PFSV, N-*ras*, K-*ras*, *rel*, *sis* and *src*. Only two oncogenes, c-*myc* and c-H-*ras*, were overexpressed in HOB1 cells (Figures 5 and 6). Other oncogenes were absent or undetectable. In addition, rearrangement of the c-*myc* gene but not the c-H-*ras* gene was observed in HOB1 cells (Figure 7).

**Figure 4** Karyotype of the HOB1 cells (partial data).

**Figure 5** Hybridisation of $^{32}$P-labelled c-*myc* or c-H-*ras* DNA to 40 µg total RNA from reactive hyperplastic lymphoid tissues (as a control) and HOB1 cell line. The ribosomal RNAs that served as size markers are indicated (28S and 18S).

**Figure 6** Hybridisation of $^{32}$P-labelled DNA probe of actin, c-H-*ras*, and c-*myc* to the indicated amounts of cellular total RNA of HOB1 and hyperplastic lymphoid tissues (as a control) respectively.

**Figure 7** Hybridisation of $^{32}$P-labelled c-*myc* DNA to the Eco I-digested cellular DNAs from HOB1 cell line and normal hyperplastic lymphoid tissues (as a control). The fragments of KpnI/SstI-digested lambda at 11 DNA were used as size marker and indicated in kb.

Chromosomal study of Burkitt's lymphoma (BL) cell line and tumours has revealed that translocation t (8:14) (q24:q32) is seen in about 90% of cases (Manalova & Manalova, 1972; Chaganti, 1983; Lenoir *et al.*, 1982; Zech *et al.*, 1976; Bernheim *et al.*, 1981), where as the translocation t (14;18) (q32;q21) is the most common translocation in non-Hodgkin's and non-Burkitt's lymphoma (Mitelman, 1980; Yunis, 1983). The t (8;14) has been particularly well studied in Burkitt's lymphoma cell lines. It has been shown to be related to molecular rearrangement of the immunoglobin genes and c-*myc* oncogenes (Berger & Bernheim, 1985; Taub *et al.*, 1982), and qualitative and quantitative abnormalities in c-*myc* expression (Stanton *et al.*, 1983; Mushinski *et al.*, 1983). This latter factor was considered to play a major role in the malignant transformation of human B lymphocyte (Hayward *et al.*, 1981; Barbacid, 1986; Nishikori *et al.*, 1984). The HOB1 cell line showed multiple chromosomal translocation, including those frequently observed in both EBV + and EBV – lymphomas. Further experiments are needed to reach a conclusion regarding a link between c-*myc* gene rearrangement and t (8;14) or other chromosomal translocations in HOB1 cells. From our data, the possible mechanism(s) of malignant conversion of the HOB1 cell line include (a) c-*myc* gene rearrangement and activation, and/ or (b) c-*myc* and c-H-*ras* genes cooperating activation. In conclusion, HOB1 is the first cell line derived from IBL with EBV – and multiple chromosomal abnormalities. It may be a useful source of cells for the study of molecular genetics in the oncogenesis of IBL and the possible role of biological agents in growth inhibition and differentiation.

This work is supported in part by the National Science Council Research Grant of Republic of China NSC77-0412-B182-10, Chang-Gung Memorial Hospital Research Fund no. CMRP244, and Institute of Biomedical Sciences, Academia Sinica, Republic of China.

### References

BAIRD, S. (1985). Antigenic markers on normal and malignant B cells. In *Monoclonal Antibodies in Cancer*, Sell, S. & Reisfeld, R. (eds) p. 147. Human Press: New Jersey.

BARBACID, M. (1986). Human oncogene. In *Important Advances in Oncology*, Devita, V.T., Hellman, S. & Rosenberg, S.A. (eds) p. 3. J.B. Lippincott: Philadelphia.

BERGER, R. & BERNHEIM, A. (1985). Cytogenetics of Burkitt's lymphoma-leukemia: a review. *IARC Sci. Publ.*, **60**, 65.

BERNHEIM, A., BERGER, R. & LENOIR, G. (1981). Cytogenetic studies on African Burkitt's lymphoma cell lines: t (8;14), t (2;8) and t (8;22) translocations. *Cancer Genet. Cytogenet.*, **3**, 307.

BHAN, A.K., NADLER, L.M., STASHENKO, P., MCCLUSKY, R.T. & SCHLOSSMAN, S.F. (1981). Stages of B cell differentiation in human lymphoid tissues. *J. Exp. Med.*, **154**, 737.

BOROWITZ, M.T., BOUSVAROS, A., BRYNES, R.K. & 5 others (1985). Monoclonal antibody phenotyping of B cell non-Hodgkin's lymphomas. The Southeastern Cancer Study Group experience. *Am. J. Pathol.*, **121**, 514.

CHAGANTI, R.S.K. (1983). Significance of chromosome change to hematopoietic neoplasms. *Blood*, **62**, 515.

CHOMCZYNSKI, P. & SACCHI, N. (1987). Single step method of RNA isolated by acid quanidinium thiocyanate-phenol-choroform extraction. *Anal. Biochem.*, **162**, 156.

DILLMAN, R.O., HANDLEY, H.H. & ROYSTON, I. (1982). Establishment and characterization of Epstein-Barr-virus-negative lymphoma B-cell line from a patient with diffuse large cell lymphoma. *Cancer Res.*, **42**, 1368.

ENGLEMAN, E.G., WARNKE, R., FOX, R.I., DILLEY, J., BENIKE, C.F. & LEVY, R. (1981). Studies of human T lymphocyte antigen recognized by a monoclonal antibody. *Proc. Natl Acad. Sci. USA*, **78**, 1791.

EPSTEIN, M.A. & BARR, Y.M. (1964). Cultivation *in vitro* of human lymphoblasts Burkitt's malignant lymphoma. *Lancet*, **i**, 525.

FREEDMAN, A.S., BOYD, A.W., ANDERSON, K.C. & 4 others (1985). Immunologic heterogeneity of diffuse large cell lymphoma. *Blood*, **65**, 630.

GODING, J.W. & BURNS, G.F. (1981). Monoclonal antibody OKT 9 recognizes the receptor for transferrin on human acute lymphocytic leukemia cells. *J. Immunol.*, **127**, 1256.

HAYWARD, W.S., NEEL, B.G. & ASTRIN, S.M. (1981). Activation of a cellular oncogene by a promoter inservation in ALV-induced lymphoid leukosis. *Nature*, **298**, 679.

HOPPE, R.T. (1982). A working formulation of non-Hodgkin's lymphomas for clinical usage: clinicopathological and prognostic correlations. In *Malignant Lymphomas*, Rosenberg, S.A. & Kaplan, H.S. (eds) p. 469. Academic Press: New York.

JANOSSY, G., BOLLUMS, F.J., BRADSTOCK, K.F. & ASHLEY, J. (1980). Cellular phenotypes of normal and leukemic hematopoietic cells determined by selected antibody combination. *Blood*, **56**, 430.

LENOIR, G.M., PREUD'HOMNE, J.L., BERNHEIM, A. & BERGER, R. (1982). Correlation between immunoglobulin light chain expression and variant translocation in Burkitt's lymphoma. *Nature*, **298**, 474.

MANALOV, G. & MANALOVA, Y. (1972). Marker band in one chromosome 14 from Burkitt's lymphoma. *Nature*, **237**, 33.

MING, P.L., TZENG, C.C. & HO, Y.S. (1987). Cytogenetic and immunologic characterization of a cell line from an extranodal immunoblast lymphoma. *Am. J. Human Genet.*, **41**, A33.

MINOWADA, J., TSUBOTA, T. & NAKAZAWA, S. (1977). Establishment and characterization of leukemic T cell lines, B cell lines, and null cell line: a progress report on surface antigen study of fresh lymphatic leukemia in man. *In Haematology and Blood Transfusion, Vol. 20*, Thierfelder, S. (ed.) p. 241. Springer-Verlag: Berlin, Heidelberg, New York.

MITELMAN, F. (1980). Marker chromosome 14+ in human cancer and leukemia. *Adv. Cancer Res.*, **34**, 141.

MOHAMED, A.N. & AL-KATIB, A. (1988). Establishment and characterization of a human lymphoma cell line (WSH-NHL) with 14; 18 translocation. *Leukemia Res.*, **12**, 833.

MUSHINSKI, J.F., BANKER, S.R., POTTER, M. & REDDY, E.P. (1983). Increased expression of myc-related oncogene mRNA characterizes most BALB/c plasmocytomas induced by pristane or Abelson murine leukemia virus. *Proc. Natl Acad. Sci. USA*, **80**, 1073.

NADLERR, L.M., STASHENKO, P., RITZ, J., HARDY, R., PESANDO, J.M. & SCHLOSSMAN, S.F. (1981). A unique cell surface antigen identifying lymphoid malignancies of B cell origin. *J. Clin. Invest.*, **67**, 134.

NILSSON, K. (1977). Establishment cell lines as tolls in the study of human lymphoma and myeloma cell characteristics. In *Haematology and Blood Transfusion, Vol. 20*, Thierfelder, S. (ed.) p. 253. Springer Verlag: Berlin, Heidelberg, New York.

NISHIKORI, M., HANSEN, H., JHANWAN, S. & 5 others (1984). Establishment of a neartertraploid B-cell lymphoma line with duplication of the 8;14 translocation. *Cancer Genet. Cytogenet.*, **12**, 39.

OMARY, M.B., TROWBRIDGE, I.S. & BATTIFORA, H.A. (1980). Human homologue of murine T200 glycoprotein. *J. Exp. Med.*, **152**, 842.

PATTENGALE, P.K., GIDLUND, M., NILSSON, K., SUNDSTROMA, C., ORN, A. & WIGZELL, H. (1981). Lysis of human B-lymphocyte-derived lymphoma/leukemia cells of established cell lines by interferon-activated natural killer (NK) cells. *Int. J. Cancer*, **28**, 459.

POPPEMA, S., BHAM, A.K., REINHERZ, E.L., MCCLUSKEY, R.T. & SCHLOSSMAN, S.F. (1981). Distribution of T cell subsets in human lymph nodes. *J. Exp. Med.*, **153**, 130.

REINHERZ, E.L., HAYNES, B.F., NADLER, L.M. & BERSTEIN, I.D. (1986). *Leukocyte Typing II*. Springer-Verlag: New York.

REINHERZ, E.L., KUNG, P.C., GOLDSTEIN, G., LEVEY, R.H. & SCHLOSSMAN, S.F. (1980). Discrete stages of human intrathymic differentiation: analysis of normal thymocytes and leukemic lymphoblasts of T-cell lineage. *Proc. Natl Acad. Sci. USA*, **77**, 1588.

RITZ, J., PESANDO, J.M., NOTIS-MCCONARTY, J., LAZARUS, H. & SCHLOSSMAN, S.F. (1980). A monoclonal antibody to human acute lymphoblastic leukemia antigen. *Nature*, **282**, 583.

STANTON, L.W., WATT, R. & MARCU, K.B. (1983). Translocation, Breakage, and truncated transcripts of c-myc oncogene in murine plasmacytomas. *Nature*, **303**, 401.

STASHENKO, P., NADLER, L.M., HARDY, R. & SCHLOSSMAN, S.F. (1980). Characterization of a human B lymphocyte-specific antigen. *J. Immunol.*, **125**, 1678.

SUNDSTROM, C. & NILSSON, K. (1978). Human malignant lymphomas *in vitro*. Characterization of biopsy cells on establishment of permanent cell lines. *Acta Pathol. Microbiol. Scand.*, **86**, 173.

TAUB, R., KIRSCH, I., MORTON, C. & 5 others (1982). Translocation of the c-myc gene into the immunoglobulin heavy chain locus in human Burkitt lymphoma and murine plasmcytoma cell. *Proc. Natl Acad. Sci. USA*, **79**, 7873.

WATANABE, S., KUROKI, M., SATO, Y., SHIMOSATO, Y. & HASEGAWA, T. (1980). The establishment of a cell line (NH-AR) from a human nodular lymphoma and a comparison with lymphoblastoid cell line. *Cancer*, **46**, 2438.

YUNIS, J.J. (1983). The chromosomal basis of human neoplasia. *Science*, **221**, 277.

ZECH, L., HAGLUND, V., NILSSON, N. & KLEIN, G. (1976). Characteristic chromosome abnormalities in biopsies and lymphoid cell lines from patients with Burkitt's and non-Burkitt's lymphoma. *Int. J. Cancer*, **17**, 47.